# Test performance metrics for breast, cervical, colon, and lung cancer screening: a systematic review

**DOI:** 10.1093/jnci/djad028

**Published:** 2023-02-08

**Authors:** Kevin Selby, Mai Sedki, Emma Levine, Aruna Kamineni, Beverly B Green, Anil Vachani, Jennifer S Haas, Debra P Ritzwoller, Jennifer M Croswell, Kabiru Ohikere, V Paul Doria-Rose, Katharine A Rendle, Jessica Chubak, Jennifer Elston Lafata, John Inadomi, Douglas A Corley

**Affiliations:** Center for Primary Care and Public Health (Unisanté), Lausanne, Switzerland; Division of Research, Kaiser Permanente Northern California, Oakland, CA, USA; University of California at San Francisco, San Francisco, CA, USA; Kaiser Permanente Washington Health Research Institute, Seattle, WA, USA; Kaiser Permanente Washington Health Research Institute, Seattle, WA, USA; Kaiser Permanente Bernard J Tyson School of Medicine, Pasadena, CA, USA; Pulmonary, Allergy, and Critical Care Division, University of Pennsylvania School of Medicine, Philadelphia, PA, USA; Division of General Internal Medicine, Massachusetts General Hospital, Boston, MA, USA; Institute for Research, Kaiser Permanente Colorado, Denver, CO, USA; Division of Cancer Control and Population Sciences, National Cancer Institute, Bethesda, MD, USA; Division of Research, Kaiser Permanente Northern California, Oakland, CA, USA; Division of Cancer Control and Population Sciences, National Cancer Institute, Bethesda, MD, USA; Department of Family Medicine & Community Health, Perelman School of Medicine, University of Pennsylvania, Philadelphia, PA, USA; Kaiser Permanente Washington Health Research Institute, Seattle, WA, USA; Eshelman School of Pharmacy and Lineberger Comprehensive Cancer Center, University of North Carolina, Chapel Hill, Chapel Hill, NC,USA; Department of Internal Medicine, University of Utah, Salt Lake City, UT, USA; Division of Research, Kaiser Permanente Northern California, Oakland, CA, USA

## Abstract

**Background:**

Multiple quality metrics have been recommended to ensure consistent, high-quality execution of screening tests for breast, cervical, colorectal, and lung cancers. However, minimal data exist evaluating the evidence base supporting these recommendations and the consistency of definitions and concepts included within and between cancer types.

**Methods:**

We performed a systematic review for each cancer type using MEDLINE, Embase, and the Cumulative Index to Nursing and Allied Health Literature (CINAHL) from 2010 to April 2020 to identify guidelines from screening programs or professional organizations containing quality metrics for tests used in breast, cervical, colorectal, and lung cancer screening. We abstracted metrics’ definitions, target performance levels, and related supporting evidence for test completeness, adequacy (sufficient visualization or collection), accuracy, and safety.

**Results:**

We identified 11 relevant guidelines with 20 suggested quality metrics for breast cancer, 5 guidelines with 9 metrics for cervical cancer, 13 guidelines with 18 metrics for colorectal cancer (CRC), and 3 guidelines with 7 metrics for lung cancer. These included 54 metrics related to adequacy (n = 6), test completeness (n = 3), accuracy (n = 33), and safety (n = 12). Target performance levels were defined for 30 metrics (56%). Ten (19%) were supported by evidence, all from breast and CRC, with no evidence cited to support metrics from cervical and lung cancer screening.

**Conclusions:**

Considerably more guideline-recommended test performance metrics exist for breast and CRC screening than cervical or lung cancer. The domains covered are inconsistent among cancers, and few targets are supported by evidence. Clearer evidence-based domains and targets are needed for test performance metrics.

**Registration:**

PROSPERO 2020 CRD42020179139

Cancer is a leading cause of morbidity and mortality in the United States and around the world (1). Population screening for asymptomatic cancer precursors and early stage breast, cervical, colorectal, and lung cancers can reduce the incidence of cervical cancer, colorectal cancer (CRC), and mortality from these 4 cancers. However, cancer screening also has potential harms (2), such as complications from procedures, anxiety from unnecessary additional testing, and overdiagnosis and resultant overtreatment (3). To maximize benefit and minimize harm, screening tests must be consistently performed using high-quality methods. Test performance quality metrics provide a potential means to monitor whether providers and programs meet defined targets and inform interventions to improve outcomes (4,5).

Quality metrics for cancer screening tests provide several opportunities: aspirational targets for individual programs, feedback to providers performing tests, criteria for competency and certification, and comparisons between different settings. Comparing quality metrics among population subgroups can reveal variation in screening effectiveness, including racial disparities (6). Useful quality metrics should provide valid and reliable measurements, be associated with meaningful outcomes, be feasible to implement, and at least partially explain unwarranted variations in clinical outcomes (7). Outcomes associated with the quality metric should either be meaningful (eg, serious injury or missed cancer) or, if the outcome is of lower impact (eg, needing to repeat an incomplete exam), potentially affect a substantial proportion of individuals screened (7). If the metric reflects a process outcome (eg, adequate bowel cleansing prior to colonoscopy), that process itself should be strongly associated with important outcomes (eg, cancer detection) and a suitable target for interventions.

Despite the widespread use of cancer screening tests and a plethora of guidelines, little work has been done to examine whether test performance quality metrics recommended in existing guidelines are evidence based and consistently defined and used across cancer types. Quality measurement and reporting place substantial burdens on providers and health-care systems (8). It is important that metrics have demonstrated benefit to support widespread implementation. Comparable metrics across cancer types allow a more consistent conceptualization of metrics, ability to identify gaps in what is measured across organ types, and comparisons among organ types for screening programs implementing multiple screening tests (9). This permits next-steps improvements and evaluation of the relative value of such improvements (10).

We conducted a systematic review to 1) identify test performance metrics recommended by professional organizations or governmental organizations for breast, cervical, colon, and lung cancer screening; 2) evaluate conceptual similarities and differences in test performance metrics across cancer types; and 3) describe levels of evidence that current guidelines utilize to support the implementation of test performance metrics. Screening includes specific tests (eg, colonoscopy) and processes (eg, consent, management of positive tests, treatment); the current evaluation focuses on test performance, defined as the technical completion of screening tests. The quality domains evaluated were created through evaluation of reported quality measures, recommended domains from the Institute of Medicine (11), and the Population-based Research to Optimize the Screening Process consortium’s quality measures working group, which included subject matter clinical and epidemiologic experts in breast, cervical, colorectal, and lung cancer screening.

## Methods

### Data sources and search strategy

This systematic review followed the Preferred Reporting Items for Systematic reviews and Meta-Analyses guidelines (registered in PROSPERO 2020 CRD42020179139). We developed search strategies with a research librarian for Ovid MEDLINE, the Cumulative Index to Nursing and Allied Health Literature (CINAHL), and Embase (Ovid) databases, using a combination of subject terms and keywords for each of the 4 cancer types: cancer type, quality metrics, and screening tests. These keywords were combined in searches using “AND” to ensure inclusion of all 3 concepts. The search was restricted to literature published in the English language from January 1, 2010, to April 6, 2020. The full search strategy is available in the Supplementary Methods (available online). Additionally, we conducted a grey literature search of bibliographies of included articles, websites of screening organizations, and recommendation documents from professional societies and governmental organizations for each cancer type.

### Study selection and eligibility criteria

Articles identified from the search were imported into an electronic database (EndNote X7.8) and systematic review management software (Covidence). Two authors independently reviewed each title and abstract for eligibility for full-text review (breast: KS, JH, and BG; cervical: AK and JCr; colorectal: MS, KS, and KO; and lung: AV and DR). Discordant review results were resolved by consensus between the 2 reviewers, with input from KS if needed. Full-text articles of eligible abstracts were retrieved and also independently reviewed for inclusion.

Our evaluation addressed quality metrics related to the technical completion of screening tests (ie, test performance). Articles reporting cancer screening test performance quality metrics for early detection of cancer or detection of precancer in average-risk, asymptomatic adults were included. We included guidelines from screening programs and professional organizations so as to emphasize consensus guidelines but not the recommendations of individuals. Each quality metric was classified within 1 of 4 domains: completeness of the test (ie, complete imaging or sampling of the target organ, such as imaging of the entire breast on mammography or visualization of the entire colon to the cecum during colonoscopy); adequacy of the examination (ie, sufficient material or visualization for a high-quality exam, such as quality of bowel preparation at colonoscopy, image quality for computerized tomography of the lung, or sufficient material from cervical sampling for Pap tests to allow cytologist interpretation); accuracy (performance characteristics such as sensitivity, specificity, false positives, etc.); and safety (ie, potential harms). We excluded guidelines primarily intended to standardize the reporting of test results, such as the American College of Radiology Breast Imaging Reporting and Data System Atlas (12). If multiple publications existed from the same recommendation body, the most recent or most complete document was used. We excluded conference abstracts, publications prior to 2010, and publications that did not develop and/or report their own quality indicators (ie, those that referred to another guideline document) or did not define quality indicators. Full descriptions of the inclusion and exclusion criteria are provided in Supplementary Materials (available online).

### Data abstraction

Three authors (KS, MS, EL) abstracted information on guideline identifiers, such as issuing body, year of publication, professional organization or society (if applicable), country of publication, conflicts of interest, funding sources if present, and quality metrics described. All test performance metrics were included from the identified guidelines; guidelines with no relevant metrics were excluded. For each quality metric, information was collected regarding the numerator and denominator definitions as well as the described level of evidence cited, if any, to support the metric. Reference standards with their inclusion and exclusion criteria were noted, when applicable. A second author out of the three (KS, MS, and EL) then reviewed abstracted data, and discrepancies were flagged for review and discussion using source documentation and consensus.

### Data synthesis and analysis

For each organ type, we summarized the total number of included guidelines, the number of organizations publishing included guidelines, number of guidelines reporting each metric, and each metric’s level of supporting evidence. Metrics were classified according to quality domain (ie, completeness, adequacy, accuracy, and safety) and number of guidelines in which they appeared.

## Results

**Figure 1.**
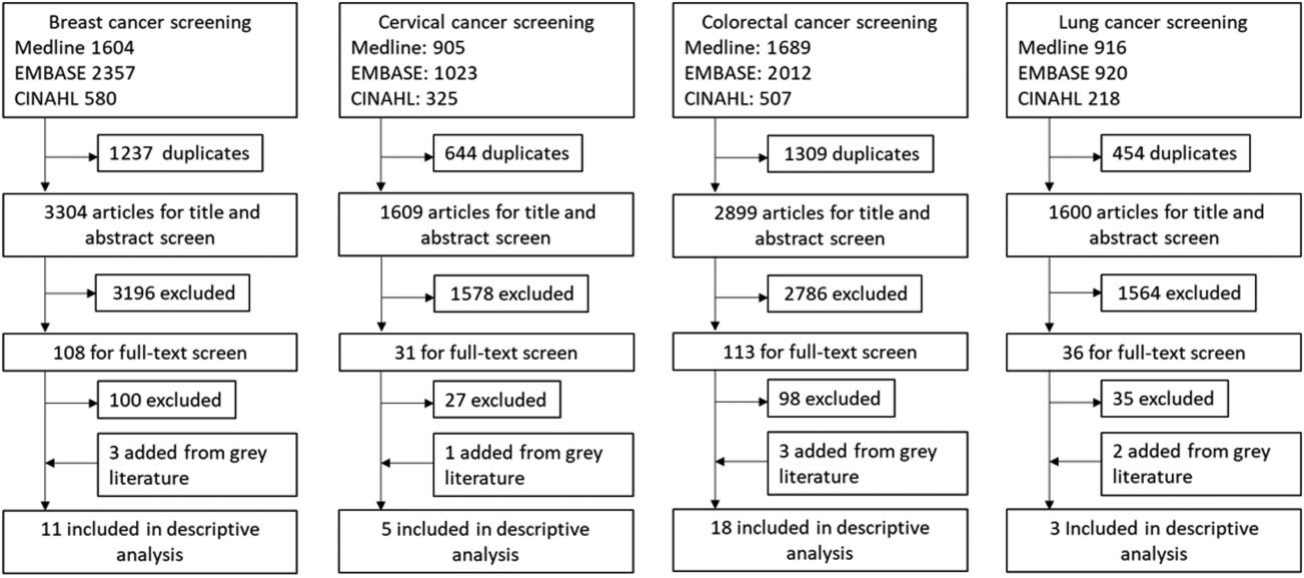
Preferred Reporting Items for Systematic Reviews and Meta-Analyses (PRISMA) flow diagram to identify articles with test performance quality metrics. Titles and abstracts were identified using search strategies for breast, cervical, colorectal, and lung cancer screening. CINAHL = Cumulative Index to Nursing and Allied Health Literature.

The database searches identified 9412 individual records from which review provided 288 relevant full texts for further evaluation by topic matter specialists (Figure 1). The main reasons for excluding full texts were that they did not provide definitions of test performance metrics, referenced other primary guideline documents rather than presenting quality metrics, or were developed by individual authors and not a screening program or professional organization. We emphasized screening programs and professional organizations to find consensus guidelines and not individual opinions. After the addition of 9 articles from the grey literature search (described above), 39 unique articles were retained from 33 organizations of which 11 were for breast cancer, 5 for cervical cancer, 14 for CRC, and 3 for lung cancer. Eighteen organizations were from Europe, 10 from North America, 4 from Asia, and 1 was multinational. Of 33 guidelines, 8 were from 2019 or later. We identified 54 individual metrics of which 20 were for breast cancer, 9 for cervical cancer, 18 for CRC, and 7 for lung cancer. Overall, 19% (n = 10) of all reported test performance quality metrics were accompanied by cited evidence beyond expert opinion, including 5 for breast cancer screening and 5 for CRC screening; cervical and lung cancer screening lacked cited supporting evidence for the listed quality metrics.

### Breast cancer screening

Eleven guidelines recommended the use of 20 different test performance quality metrics for breast cancer screening of which 2 addressed test adequacy, 15 addressed test accuracy, and 3 addressed test safety (Table 1;Supplementary Table 1, available online). The 5 most frequently reported metrics, all of which measure test accuracy, were the recall rate (11 of 11 guidelines), defined as the proportion of mammograms read as requiring repeat or further assessment, and cancer detection rate (10 of 11 guidelines), defined as the number of cancers detected per 1000 women screened, followed by cancer size, interval cancer rate, and the positive predictive value of positive mammograms (all in 7 of 11 guidelines). Of 20 metrics, 5 were referenced as supported by evidence beyond expert opinion in at least 1 guideline. For instance, the recall or call-rate targets are supported by observational studies demonstrating upper limits above which additional positive tests cause more harm than benefit (13). Metrics measuring outcomes of direct importance to patients, such as the interval cancer rate, were supported by data from multiple programs demonstrating the feasibility of interval cancer rates below a target performance level. Target performance levels were provided for 13 of 20 metrics, most often with acceptable and desirable levels of performance.

**Table 1.** Twenty test performance quality metrics for mammographic breast cancer screening from 11 organizations

Quality domain (number per category)	Metric (number of organizations): definition	Target level of performance
Completeness (n=0)		
Adequacy (n=2)	Technical repeats and recalls (2 organizations): % exams requiring repeat because the first image is inadequate (37,38)	AL <2% or 3%; DL <1% (37,38)
	Image quality (1 organization): thickness measured using a test object (37)	Varies by test object. For 1 mm test object, AL <0.091 mm (37)
Accuracy (n=15)	**Recall rate (11 organizations): % Breast Imaging Reporting and Data System (BI-RADS) 0, 4, or 5 recalled for further assessment per persons screened (13,29,37-45)	Prevalent: AL <10%; DL <7 %; incident: AL <7%; DL <5% (13,37,38,40); or range ≥5% to ≤14% (44)
	**Cancer detection rate (10 organizations): number of (invasive) cancers per 1000 screened (13,29,37-39,41-45)	Prevalent: >5 per 1000; incident: >2.4 (38) or >3 per 1000 (13,44)^b^
	Cancer size (7 organizations): invasive cancers ≤10 mm or 15 mm as % of all invasive cancers (13,29,37,38,40,42,44)	≥25% cancers ≤10 mm; ≥50% cancers ≤15 mm (13,37,40)
	**Interval cancer rate (7 organizations): interval cancers per 1000 screened (13,29,37-40,42)	<0.75 per 1000 at 0-12 mo; <1.25 per 1000 at 12-24 mo (13,38)
	**Positive predictive value (7 organizations): % cancers per positive mammograms (13,38,39,41,43-45)	Prevalent: ≥5%; incident: ≥6% (13); or range ≥3% to ≤8% (44)
	**Program sensitivity (6 organizations): screen detected cancers per all cancers detected before next scheduled exam (1 to 2 y) (13,29,39,40,42,43,45)	>70% at 12 mo; >50% from 12 to 24 mo (40)
	Localized cancers (6 organizations): node-negative cancers per screen-detected cancers (13,29,37,39,40,44)	Prevalent: >70%; incident: >75% (13,40)^c^
	DCIS (5 organizations): % DCIS/cancers (13,38-40)	10%-20% (38,40)
	Program specificity (3 organizations): % women without cancer detected before the next scheduled exam who had a normal mammography (39,42,45)	
	False-negative rate (2 organizations): false negative per 1000 screens (43,45)	
	Early recall (2 organizations): % recalled at interval shorter than normal (37,38)	<1% (37,38)
	False-positive recall rate (1 organization): recalls not followed by cancer per 1000 screened (39)	
	Stage ≥II cancers (1 organization): stage ≥II cancers per screen-detected cancers (39)	
	Third reading (1 organization): % exams needing third reading/all screens (39)	
	Consistency in diagnosis (1 organization): diagnosis consistency (38)	Kappa >0.8 (38)
Safety (n=3)	Benign open biopsy or surgery rate (4 organizations): benign biopsies or surgeries per 1000 screens (13,29,37,38,40)	Prevalent: <3.6 per 1000; incident: <2.0 per 1000 (37)
	Radiation dose: for a standard mammogram (37,40)	<2.0 or <2.5 mGy (37,40)
	Mastectomy rate (1 organization): women with mastectomy per 1000 screened (29)	

a Metrics preceded by ** cited supporting evidence beyond expert opinion. Complete abstracted data are available in Supplementary Table 1 (available online). AL = acceptable level; DL = desirable level; DCIS = ductal carcinoma in situ.

b Alternatively the age-standardized cancer ratios for invasive cancers: >1.0 per 1000 (37).

c Alternative target: >50% in situ or stage I (44).

### Cervical cancer screening

Five guidelines recommended 9 test performance quality metrics for cervical cancer screening of which 7 addressed test accuracy and 2 addressed test adequacy (Table 2;Supplementary Table 2, available online). Of 5 guidelines, 4 included specimen adequacy (either as the portion of all tests judged to be adequate or criteria for individual tests), and 3 of 5 included the positive predictive value of abnormal screening results and the precancer detection rate. No guidelines provided evidence to support the recommended test performance metrics, and 3 of 9 metrics had target performance levels, though these also lacked citations for an evidence base.

**Table 2.** Nine test performance quality metrics for cervical cancer screening from 5 organizations^a^

Category (number per category)	Metric (number of organizations): definition	Target level of performance
Completeness (n=0)		
Adequacy (n=2)	Specimen adequacy (3 organizations): % of specimens deemed inadequate or unsatisfactory (46-48)	0.5%-2% of tests inadequate (48)
	Individual specimen adequacy (1 organization): specimens should have ≥5000 squamous cells, with ≤75% of squamous cells obscured to be considered adequate, provided no abnormal cells are identified (36)	
Accuracy (n=7)	Positive predictive value of abnormal screening results (3 organizations): % of abnormal results with histologically confirmed CIN+ (defined as CIN2 or more severe) (46,47,49)	
	Precancer detection rate (3 organizations): number of detected precancerous lesions per 1000 women screened (46,48,49)	
	Interval cancers (2 organizations): % cancer diagnosed between 0.5 and 3 years after a negative cytology test (48,49)	
	Sensitivity (1 organization): % first cytology examination read as abnormal/first read abnormal and/or abnormals identified at rapid review (47)	>90% for all abnormalities; >95% for high-grade abnormalities (47)
	Cytology–histology agreement (1 organization): % of high-grade cytology tests with an abnormal histological outcome (48)	≥65% (48)
	Early stage cancers (1 organization): % of cancers detected at stage I per total cancers detected (48)	
	Test specificity (1 organization): % screened women not referred for colposcopy/screened women without histologically confirmed CIN+ (49)	
Safety (n=0)		

a Quality metrics apply to cytology-based and human papillomavirus–based screening, unless specified. No metrics cited evidence beyond expert opinion. Targets are provided when available. Full extraction is available in Supplementary Table 2 (available online). CIN = cervical intraepithelial neoplasia.

### Colorectal cancer screening

Fourteen organizations recommended 18 test performance quality metrics for CRC screening of which 2 addressed test adequacy, 3 test completeness, 8 test accuracy, and 5 test safety (Table 3;Supplementary Table 3, available online). The 3 most frequently recommended metrics, all measuring colonoscopy performance, were the cecal intubation rate (11 of 14 guidelines), adenoma detection rate (10 of 14 guidelines), and scope withdrawal time (7 of 14 guidelines). The inadequacy rate, defined as the proportion of fecal occult blood tests that cannot be processed and therefore needs to be repeated, was the most frequently recommended metric for fecal occult blood test quality (6 of 14 guidelines). Five metrics were supported by observational studies demonstrating a benefit to better performance. For instance, higher adenoma detection rates were associated with reduced postcolonoscopy cancer rates, and longer withdrawal times and better bowel preparation were linked to higher adenoma detection rates. Target performance levels were provided for 14 of 18 metrics.

**Table 3.** Eighteen test performance quality metrics stool- or colonoscopy-based colorectal cancer (CRC) screening from 14 organizations. Thirteen metrics are for colonoscopy-performance alone, 3 for stool testing-performance alone, and 2 for either type of screening^a^

Category (number per category)	Metric (number of organizations): definition	Target level of performance
Complete-ness (n=3)	**Cecal intubation rate (11 organizations): % of colonoscopies with photo taken of cecum (50-60)	AL >90%; DL ≥95% (50-56,58,59); or AL ≥92%; DL ≥97% (51)
	**Scope withdrawal time (8 organizations): average time (min) to withdraw colonoscope when outcome is normal (51,53,55-59,61)	AL ≥6 min (51,53,55-58); DL ≥10 min (51)
	Polyp removal/retrieval (4 organizations): % polyps sent for pathology per all polyps (51,53,54,56)	AL >90% polyps (51,53,56); DL >95% (51)
Adequacy (n=2)	**Adequate bowel preparation (6 organizations): % adequate per all screening colonoscopies (53,54,56,57,59,61)	≥85% (57,58) or >90% (53,54,56) of exams
	FOBT inadequacy rate (6 organizations): % inadequate FOBT per persons tested (50,52,54,60,62,63)	AL <3% (50) or <5% (63); DL <1% (50,52)
Accuracy (n=8)	**Adenoma detection rate (10 organizations): number with adenomas/screening colonoscopies (50,51,53-56,59,60,62,63)	≥15% (56), ≥20% (53,55), or ≥25% (58,59)^b^
	High-grade neoplasia reported (1 organization): % of biopsies with high-grade neoplasia (50)	<5% screening colonoscopies; <10% after positive FOBT (50)
	Postcolonoscopy CRC rate (1 organization): % of all CRCs diagnosed after a negative colonoscopy (56)	<5% at 3-year follow-up after colonoscopy (56)
	Polyp detection rate (1 organization): number with polyps per screening colonoscopies (62)	
	FOBT positivity rate (4 organizations): % positive FOBT per tested population (52,54,62,63)	Prevalence round: AL <6%; DL <5% (52)
	FOBT positive predictive value (1 organization): % positive fecal tests with an advanced adenoma or CRC (52)	>50% for FIT; >25% (52) or >35% for gFOBT (63)
	Cancer detection rate (4 organizations): screen detected cancers per number of people screened (52,54,62,63)	AL >2 per 1000; DL >2.5 per 1000 (52,63)
	CRC stage distribution (3 organizations): % CRC diagnosed at stage III-IV (or the inverse) (52,54,63)	AL <30%; DL <20% (52)
Safety (n=5)	**Rate of perforation (4 organizations): % endoscopies with perforation (53,55,56,58,59)	<1 per 1000 (55,58,59)
	**Postpolypectomy bleeding rate (4 organizations): % colonoscopies with clinically significant bleeding postpolypectomy (53,55,56,58,59)	<1 per 100 (58,59); <1 per 200 (55)
	Adverse or unplanned events after colonoscopy (4 organizations): % colonoscopies with adverse event within 30 days (53,54,58,61)	
	Mortality (1 organization): 30-day all-cause and colonoscopy-related mortality/tested population (62)	
	Comfort score (1 organization): % colonoscopies with moderate-severe patient discomfort (53)	

a Metrics preceded by ** cited evidence beyond expert opinion in at least 1 guideline. Targets are provided when available. Full extraction is available in Supplementary Table 3 (available online).

b Adenoma detection rate of ≥40% recommended after a positive FIT (51,55). AL = acceptable limit; CRC = colorectal cancer; DL = desirable limit; FIT = fecal immunochemical test; FOBT = fecal occult blood tests (guaiac [gFOBT] and immunochemical).

### Lung cancer screening

Three organizations recommended 7 test performance quality metrics for lung cancer screening of which 6 came from 1 organization (14) (Table 4;Supplementary Table 4, available online). Three of these metrics relate to test adequacy and 4 to test safety. The most frequently reported metric was radiation dose from low-dose computed tomography (LDCT) (a reflection of test safety), appearing in 3 of 4 guidelines. No guidelines provided specific evidence to support the recommended metrics for LDCT test performance, and only 1 of 7 recommended a target level.

**Table 4.** Seven test performance quality metrics for low-dose computed tomography (LDCT) lung cancer screening from 3 organizations^a^

Category (number per category)	Metric (number of organizations): definition	Target level of performance
Completeness (n=0)		
Adequacy (n=0)		
Accuracy (n=3)	Early reassessment index (1 organization): % screens requiring any additional test or referral prior to the next routinely scheduled screen (14)	
	Positive predictive value (1 organization): % diagnosed with lung cancer among those requiring early reassessment (14)	
	Cancer detection rate (1 organization): cancers per 1000 in program or among 1000 biopsied (14)	
Safety (n=4)	Radiation dose (2 organization): effective dose (64,65)	1-3 milli-sieverts (64,65)
	Invasive procedure rate (1 organization): % participants who undergo an invasive procedure (14)	
	Nonmalignant surgical biopsy or resection rate (1 organization): number of surgical lung biopsies or resections with a nonmalignant result per 1000 screens (14)	
	30-day mortality rate after surgical procedure (1 organization): % who died within 30 days among participants who underwent a surgical procedure (14)	

a Definitions regroup common points from the listed articles. No metrics cited evidence beyond expert opinion. Targets are provided when available. Full extraction is available in Supplementary Table 4 (available online)

## Discussion

This systematic review of test performance quality metrics for 4 cancer screening processes revealed multiple guideline-recommended quality metrics for breast and CRC screening and relatively few for cervical and lung cancer screening. Only a minority of the recommended metrics were supported by evidence. More than half of recommended metrics measured test accuracy at the level of individual providers or entire programs, with fewer metrics measuring safety, adequacy, and test completeness. The types of metrics used varied considerably between cancer types. When provided, the evidence to support quality metrics and target performance thresholds was derived from either demonstration of associations between test performance variation and cancer outcomes or attainable performance levels from high-quality screening programs.

The current study compares existing test performance quality metrics within and across organ types; the higher number of evidence-based metrics for breast and CRC screening reveals potential gaps for lung and cervical cancers. Lung cancer screening with LDCT has been implemented more recently; thus, there are fewer large-scale, organized programs to collect test performance data, likely explaining the fewer metrics for LDCT. Moreover, relative to the other 3 organ-specific cancer screening services, currently no National Committee for Quality Assurance or Healthcare Effectiveness Data and Information Set reporting measures exist for lung cancer screening (15). Although cervical cancer testing was first recommended nearly 70 years ago, only 9 quality metrics were identified. The Bethesda System for Reporting Cervical Cytology provides minimum benchmarks for test adequacy; these were included in Table 2 of the current publication even if the Bethesda System does not fully address other standards such as accuracy and safety nor, as a system mainly aimed at reporting, does it focus on specimen collection methods for minimizing the proportion of tests that are inadequate, which is typically the primary focus of quality metrics aimed at adequacy (13). Further, the proportion of specimens that are inadequate has been inconsistently linked to higher rates of neoplasia in studies now performed more than 20 years ago (16,17). These studies were not cited by guidelines to support the use of the quality metric. The lack of subsequent studies may be because cervical cancer screening is a simple, relatively noninvasive, and well-established practice; although screening indications and intervals have changed frequently, test performance has remained fairly stable (18). With the widespread adoption of commercially available human papillomavirus tests, some of the work of monitoring cervical cancer screening test performance has been assumed by commercial entities and the Food and Drug Administration. The Food and Drug Administration rigorously assesses the reproducibility and performance of all tests before approval and requires ongoing quality assurance. The relatively minor harms associated with cervical cancer screening (discomfort or bleeding), coupled with its remarkable success in reducing cervical cancer incidence and mortality, may have resulted in less attention to quality measurement in test performance (19). However, data from 427 clinics in New Mexico demonstrated a greater than twofold difference in screening positivity rate between clinics at the 25th and 75th percentile of volume (approximately 55 to 115 of 1000 cytology smears read as abnormal)—far greater than observed between clinics performing breast cancer and CRC screening (20). It is not known whether those differences are driven by underlying population risk vs unwarranted variations in care with potential clinical impact, positive or negative. Documenting variation is a key first step for establishing metrics, which may improve consistency and effectiveness (21).

**Figure 2.**
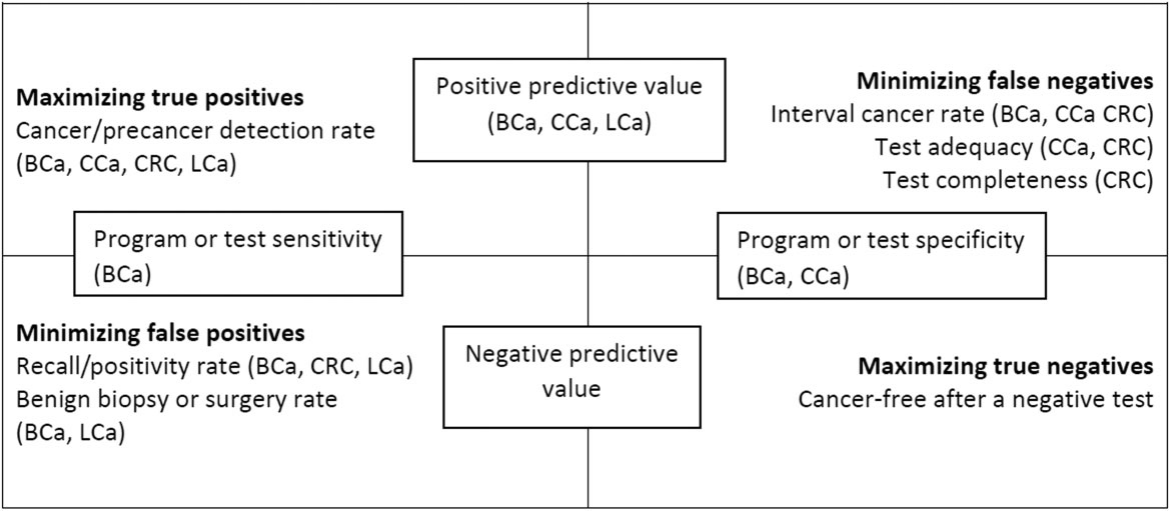
Objectives of high-quality screening and corresponding test performance quality metrics. Metrics in brackets without specified organ types are not currently suggested in included guidelines. BCa = breast cancer; CCa = cervical cancer; CRC = colorectal cancer; LCa = lung cancer.

Measuring and reporting some quality metrics may be more useful for some cancer types or screening tests than others; for example, lung LDCTs are more than 99% complete (the entire lung is visualized) and adequate (eg, the quality of the image is sufficient); this suggests routinely measuring and reporting quality metrics in this domain may be of low value, provided excessive radiation doses are not being used to give such high rates of adequate studies (22). Identified test accuracy metrics were more similar across cancers than measures of adequacy, completeness, and safety. Figure 2 provides a conceptual overview of test performance metrics. An important difference between CRC screening tests and others is that there has been relatively less emphasis on false-positive colonoscopies (ie, a colonoscopy with biopsy or reporting of a clinically insignificant lesion). This difference is likely because until now there has been no upper limit for adenoma detection rate above which additional detection is harmful, nor a consensus definition of overdiagnosis (23). Conversely, breast cancer screening aims for a balance between increasing the cancer detection rate (cancers per 1000 women participating) and decreasing the recall rate (proportion of mammograms read as requiring further assessment); although increasing the recall rate reduces interval cancers, there appears to be a ceiling above which a higher recall rate is primarily linked to false-positives and overdiagnosis (24).

Which test accuracy domains will be most important to the balance of risks and benefits of lung cancer screening remains to be determined. A recent initiative of the National Lung Cancer Roundtable has identified quality indicators for lung cancer screening. However, those reaching consensus were process indicators (ie, screening rates and follow-up time) and not test performance indicators. The proportion of nonsurgical and surgical biopsies with benign results (false-positive rate) and the proportion of stage I cancers were identified as highly relevant but eventually not retained because important data were not available at the time in the American College of Radiology Lung Cancer Screening Registry (25). No guidelines recommend reporting the proportion of true-negatives or negative predictive value (Figure 2), possibly because it would take several years of cancer-free follow-up to demonstrate true-negative status.

Quality metrics may require substantial provider and health system effort to measure and to modify, yet few of the quality metrics identified cited supporting evidence for their use. Ideally, readily measurable test performance metrics linked to outcomes of importance should be accurately measured and available for all screening tests. Conversely, quality metrics without a demonstrated impact or utility could be discarded. An example of a successfully developed, readily available, validated, and implemented quality metric is the adenoma detection rate for screening colonoscopy. This measures, at the level of individual providers, the percentage of screening exams that detect at least 1 precancerous (ie, adenomatous) polyp. For example, if a provider performs screening colonoscopies for 100 patients, and 35 of these exams detect at least 1 adenoma, the provider’s adenoma detection rate is 35%. Each 1% decrease in adenoma detection rate has been associated with a 3%-4% increase in postcolonoscopy cancer risk and with increases in risk of cancer-related mortality (26). It can be calculated regularly for individual providers, and training interventions can lead to improvements in the detection rate that are associated, in turn, with improvements in patient outcomes (27). Improvements in some other CRC screening metrics, such as exam completeness (cecal intubation rate) and adequacy (bowel preparation), are also associated with increases in adenoma detection and postcolonoscopy CRCs. Such evidence is sorely lacking not only for cervical and lung cancer screening metrics but also for some widely used metrics for breast cancer. Large databases with diverse populations and screening centers, large numbers of detailed screening test reports, and comprehensive tracking of participant outcomes such as cancer incidence and mortality could provide an opportunity to strengthen research in this area (28). Interventional studies are also needed to demonstrate the ability to improve performance on quality metrics; some metrics may be intuitively important (eg, mortality rate of screening-related procedures), without being feasible targets for improvement.

Previous research has attempted to describe and prioritize quality metrics, primarily for individual cancer screening tests (29). To our knowledge, this study is the first effort to comprehensively identify all available screening test quality metrics across the 4 cancers for which screening is recommended in the United States. A prior Delphi consensus process with experts in breast, cervical, and colorectal cancers identified 10 conceptual measures for screening test quality, including 5 outcomes for potentially setting targets related to test performance: interval cancer rate, detection rate, cancer incidence, cause-specific mortality, and distribution of cancers by mode of detection (30). Quality measures ideally would include those amenable to rapid feedback to individual performing clinicians and long-term measures, such as cancer rates, for informing broader decisions about population-based screening programs. However, most existing measures require substantial passage of time to measure, linkage to a cancer registry, and reporting at the program level, as opposed to feedback to individual clinicians. Performance metrics requiring linkage to cancer outcomes and with very low incidence, such as colonoscopy-related mortality, are inherently more challenging to meaningfully implement. Ideally, quality metrics should be practical to measure and usable for monitoring screening quality and/or to improve care—ideally in as close to real-time as possible, such as the demonstration of associations between the recall rate for mammography and postscreening cancer rates (31). For example, individual radiologists, provided they read more than 2700 mammograms per year, can receive robust estimates of their performance as compared with established benchmarks, though implementation in the United States remains incomplete (32). If done properly and not overused, audit and feedback of health professionals can dramatically improve care (33). Conversely, measuring and tracking quality metrics require important resources (34); thus, they should predict important outcomes and ideally inform modifiable changes so that “the aim should be to measure only what matters, and mainly for learning” (35). Further research is urgently needed to better prioritize between metrics.

Strengths of this review include the comprehensive searches performed simultaneously across multiple cancers, efforts to search the grey literature, and the participation of topic experts in all included cancer types. There are also some weaknesses. We may have underascertained the evidence available to support the included quality metrics as we assumed that guidelines would cite available evidence supporting the metric. Quality metrics for screening programs are sometimes published uniquely in the local language, such that some from non-English guidelines may have been missed. Most quality metrics came from organized national screening programs and may not be generalizable to the US context of primarily opportunistic screening. Finally, some relevant guidelines may have been published prior to 2010 and thus missed by our database search. However, we searched bibliographies of included literature and the grey literature; presumably these guidelines, if still used, would have been cited by more recent literature. We included some guidance documents, such as the Bethesda System, that primarily standardized the reporting of test results, for their relevant sections that addressed concepts regarding specimen adequacy (36).

In conclusion, in this comprehensive, cross-cancer, systematic evaluation of quality metrics for cancer screening test performance, we identified substantial gaps in the existence of quality metrics, notably for cervical and lung cancers, in the evidence available to support use of existing metrics for performance monitoring and quality improvement, and in conceptual consistency of quality domains across cancer types. Further work is needed to determine which test performance quality domains can best be used to monitor and improve cancer screening and which existing performance metrics can be safely discarded.

## Supplementary Material

djad028_Supplementary_Data

## Data Availability

All collected data are available upon request.
