# Supplementary material for: Test performance metrics for breast, cervical, colon, and lung cancer screening: a systematic review
Source: J Natl Cancer Inst. 2023 Feb 8;115(4):375–84. doi: 10.1093/jnci/djad028 (PMC10086636; doi:10.1093/jnci/djad028)
Supplement: djad028_Supplementary_Data [file djad028_supplementary_data.pdf]

Supplementary material

Section Index

Supplementary Methods: Search terms and strategy 2
Colorectal cancer: 2
Cervical cancer 3
Lung cancer: 4
Breast cancer: 5
Supplementary Results: Full extraction tables by cancer type 7
Supplementary Table 1: Breast cancer 7
Supplementary Table 2: Cervical cancer 10
Supplementary Table 3: Colorectal cancer 12
Supplementary Table 4: Lung cancer 15

Supplementary Methods: Search terms and strategy

Information sources: MEDLINE, Embase and CINAHL

Colorectal cancer:

(PMIDs for testing with CRC: 25448873, 30052593, 23212726, 31637887, 21792895, https://www.gov.uk/government/publications/bowel-cancer-screening-programme-standards/bowel-cancer-screening-programme-standards-valid-for-data-collected-from-1-april-2018)

Colorectal cancer search April 6th:

Databases and web portals	Date	Number of references found	Number of references after deduplication
Medline OVID SP	6-Apr-20	1689	1685
Embase.com	6-Apr-20	2012	1105
CINAHL EBSCO	06.04.20	507	114
Total		4208	2904

Concept map (#1 AND #2 AND #3):

1. Quality indicators - ((('health care quality'/de OR 'benchmarking'/de OR 'clinical indicator'/de OR 'practice guideline'/exp OR 'program evaluation'/exp OR 'professional standard'/de OR 'treatment outcome'/exp OR 'quality control'/de OR 'total quality management'/de OR 'treatment outcome*':ti,ab,kw) AND (assessment* or criteri* or guideline* or indicator* or metrics or recommendation*):ti,ab,kw) OR ((quality NEAR/2 (assessment* or criteri* or guideline* or 'health care' or healthcare or indicator* or metrics or recommendation*)) or 'performance indicator*' or 'process assessment*'):ti,ab,kw)

AND

2. Cancer type - ('colon tumor'/exp OR 'large intestine cancer'/de OR 'rectum tumor'/exp OR 'intestine polyp'/de OR 'colorectal polyp'/de OR ('colonoscopy'/de AND indicator*:ti,ab) OR (((colon or colonic or colorectal or colo-rectal) NEAR/2 (cancer* or carcinoma or neoplas* or polyp*)) or intestinal polyp*):ti,ab,kw)

AND

3. Screening tests - ('colonoscopy'/exp OR 'occult blood test'/de OR 'early cancer diagnosis'/de OR 'cancer screening'/mj OR ('colonoscopy' OR 'coloscopy' OR 'faecal immunochemical test*' OR 'fecal immunochemical test*' OR faecal occult blood test* OR fecal occult blood test*):ti,ab,kw)

Additional criteria:

[english]/lim AND [2001-2020]/py NOT ([animals]/lim NOT [humans]/lim) NOT ('conference abstract'/it OR 'conference review'/it OR 'editorial'/it OR 'letter'/it)

Additional data sources: Search of bibliographies, websites of screening organizations, asking experts

Study selection criteria: To determine eligibility, two reviewers will independently screen the title and abstract of each identified guideline article. To be included, guidelines must meet the following criteria:

- Report quality criteria for the early detection of cancer in average risk, asymptomatic adults
- Examine screening tests in one of four domains: **completeness** of the test (what defines a complete test with adequate sampling as estimated by the clinical provider obtaining the specimen); **adequacy** of the examination (what defines sufficient material or visualization for a high quality exam); **accuracy** (performance characteristics such as sensitivity, specificity, false positive rate, etc.); **safety** (what are potential harms).
- Address breast, cervical, colon or lung cancer screening
- Be a recommendation document from a professional society, screening organization, or governmental organization
- Can be a performance report from a national or regional screening program, provided three are clear definitions of quality indicators and set targets
- Have sufficient English-language content to permit extraction of all quality indicators

Exclusion criteria are:

- Conference abstracts
- Publications from 2009 or before
- Quality criteria development and validation documents
- Performance reports that do not have their own quality indicators (i.e. refer to another guideline document), do not define quality indicators, or do not set targets

Cervical cancer

Example PMIDs: 20176693, 25817010, 29394915, 31106635
Example website: Breast, cervical and CRC : https://www.cdc.gov/cancer/nbccedp/pdf/measuring-cancer-screening-rates-508.pdf

MEDLINE March 5th test search: 896 articles

1. Quality indicators - (exp "Quality Indicators, Health Care"/ or (("Quality of Health Care"/ or exp "Outcome and Process Assessment, Health Care"/ or exp Program Evaluation/ or exp Quality Assurance, Health Care/ or Mass Screening/mt, st or Reference Standards/ or Quality Control/ or treatment outcome*.ti,ab,kf.) AND (assessment* or criteri* or guideline* or indicator* or metrics or recommendation*).ti,ab,kf.) or ((quality adj2 (assessment* or criteri* or guideline* or "health care" or healthcare or indicator* or metrics or recommendation*)) or performance indicator* or process assessment*).ti,ab,kf.)

2. Cancer type - (Uterine Cervical Neoplasms/ or exp Uterine Cervical Dysplasia / or Cervical Intraepithelial Neoplasia/or ((Papanicolaou Test/ or Vaginal smears/) and indicator*.ti,ab.) or ((cervix or cervical) adj2 (cancer* or carcinoma or neoplas*)).ti,ab,kf.)

3. Screening - ("Early Detection of Cancer"/ or "Mass Screening"/ or Papanicolaou Test/ or Human Papillomavirus DNA Tests/ or Vaginal smears/ OR (Papanicolaou test* or pap test* or pap smear* or HPV test* or Human papilloma virus test* or vaginal smear*).ti,ab,kf.)

Additional criteria:
English.lg. NOT (exp animals/ not humans/) NOT (editorial/ or letter/)
limit to yr="2009 -Current"

Lung cancer:

Example PMIDs: 25356819, 26426785,
Example websites: https://www.england.nhs.uk/wp-content/uploads/2019/02/targeted-screening-for-lung-cancer-quality-assurance-standard.pdf; https://www.acr.org/-/media/ACR/Files/Practice-Parameters/CT-LungCaScr.pdf?la=en

March 4th test MEDLINE: 902 references found

1. Quality indicators - (exp "Quality Indicators, Health Care"/ or (("Quality of Health Care"/ or exp "Outcome and Process Assessment, Health Care"/ or exp Program Evaluation/ or exp Quality Assurance, Health Care/ or Mass Screening/mt, st or Reference Standards/ or Quality Control/ or treatment outcome*.ti,ab,kf.) AND (assessment* or criteri* or guideline* or indicator* or metrics or recommendation*).ti,ab,kf.) or ((quality adj2 (assessment* or criteri* or guideline* or "health care" or healthcare or indicator* or metrics or recommendation*)) or performance indicator* or process assessment*).ti,ab,kf.)

2. Cancer type - (exp Lung Neoplasms/ or Carcinoma, Small Cell/ or Carcinoma, Non-Small-Cell Lung/or exp Adenocarcinoma of Lung/ or ((lung or pulmonary or bronch*) adj2 (cancer* or carcinoma or neoplas*)).ti,ab,kf.)

3. Screening - ("Early Detection of Cancer"/ or "Mass Screening"/ OR exp Tomography, X-Ray Computed/ or (low-dose computed tomography or computed tomography or low-dose CT or LDCT).ti,ab,kf.)

Additional criteria:
English.lg. NOT (exp animals/ not humans/) NOT (editorial/ or letter/)

limit to yr="2009 -Current"

Breast cancer:

Example PMIDs: 18024988; 26405774; 24261339; 21554747; 31660890 (no MeSH keywords yet)
Example websites: https://qpp.cms.gov/docs/QPP_quality_measure_specifications/CQM-Measures/2019_Measure_112_MIPSCQM.pdf (NCQA); https://www.cdc.gov/cancer/nbccedp/pdf/measuring-cancer-screening-rates-508.pdf; https://www.acr.org/Clinical-Resources/Reporting-and-Data-Systems/Bi-Rads; https://www.gov.uk/government/publications/breast-screening-consolidated-programme-standards/nhs-breast-screening-programme-screening-standards-valid-for-data-collected-from-1-april-2017; Breast, cervical and CRC : https://www.cdc.gov/cancer/nbccedp/pdf/measuring-cancer-screening-rates-508.pdf

March 4th test MEDLINE search: 1591 titles identified

1. Quality indicators - (exp "Quality Indicators, Health Care"/ or (("Quality of Health Care"/ or exp "Outcome and Process Assessment, Health Care"/ or exp Program Evaluation/ or exp Quality Assurance, Health Care/ or Mass Screening/mt, st or Reference Standards/ or Quality Control/ or treatment outcome*.ti,ab,kf.) AND (assessment* or criteri* or guideline* or indicator* or metrics or recommendation*).ti,ab,kf.) or ((quality adj2 (assessment* or criteri* or guideline* or "health care" or healthcare or indicator* or metrics or recommendation*)) or performance indicator* or process assessment*).ti,ab,kf.)

2. Cancer type - (exp Breast Neoplasms/ or Breast Carcinoma In Situ/ or (exp Mammography/ and indicator*.ti,ab.) or ((breast or mammary) adj2 (cancer* or carcinoma or neoplas*)).ti,ab,kf.)

3. Screening - (*"Early Detection of Cancer"/ or *"Mass Screening"/ or exp Mammography/ or Magnetic resonance imaging/ or (mammography or digital mammography or magnetic resonance imaging* or MRI*).ti,ab,kf.)

Additional criteria:
English.lg. NOT (exp animals/ not humans/) NOT (editorial/ or letter/)

limit to yr="2009 -Current"

Supplementary Results: Full extraction tables by cancer type

Supplementary Table 1: Breast cancer

Twenty quality indicators from 11 organizations identified for breast cancer screening with mammography, listed by continent and organization name. Definitions regroup common points from the listed articles, with important divergences noted. Each ‘X’ indicates that the quality indicator is present in that article with a similar definition to that listed. Level of evidence and targets are provided when available.

Category (number per category)	Indicator – definition (number of organizations using this indicator)	Level of evidence (Reported for 7/20 indicators)	Target (Reported for 13/20 indicators)	Organization, Year of publication (n=11)										
				European Commission Initiative on Breast Cancer, 2021(1)	Belgium – Flemish screening programme, 2014 & 2019(2)	Denmark – Danish Quality database, 2013(3)	England - NHS Breast Screening program, 2018(4) & 2021(5)	Ireland - BreastCheck Programme, 2018(6) & 2015(7)	Italy – Italian screening group, 2015(8)	Singapore – National Screening Programme, 2015(9)	Taiwan – National Screening Program, 2011(10)	Canada – Organized screening programs, 2013(11)	USA, Chicago Breast Cancer Quality Consortium, 2014(12)	USA, Breast Cancer Surveillance Consortium, 2017(13)
Adequancy (2)	**Technical repeats and re-calls** - % exams requiring repeat for technical reasons (2)		AL: <3%, DL: <1%											
	**Image quality** – Thickness measured using a test object (1)		Varies based on diameter of detail. For 1 mm, AL <0.091 mm											
Accuracy (14)	**Recall rate** - % BIRADS 0, 4 or 5 recalled for further assessment / screened (11)	Obs. Studies, int. comparison	Prevalent: AL < 10%, DL < 7 %; Incident: AL < 7%, DL < 5%										Any recall and biopsy rec. rate	
	**Cancer detection rate** – number of (invasive) cancers / 1000 screened (10)	Obs. Studies, int. comparison	Prevalent: >5/1,000 Incident: >3/1,000											

	**Cancer size -** invasive cancers ≤ 10mm or 15mm as % of all invasive cancers (7)	Int. comparison	Prevalent: ≥25% cancers ≤ 10mm; Incident: ≥50% cancers ≤ 15mm	Size ≤10mm & >20 mm										
	**Interval cancer rate** – interval cancers / 1000 screened (7)	Obs. Studies and int. comparison	<0.75/1,000 at 0-12 months <1.25 per 1,000 at 12-24 months									Postscreen invasive cancer rate		
	**Positive predictive value** - % cancers / positive mammograms (7)	Obs. Studies and int. comparison	Prevalent: ≥5%; Incident: ≥ 6%										PPV of mammos and of biopsies	PPV of biopsy rec. and of biopsies
	**Programme sensitivity** – screen detected cancers / all cancers (6)	Obs. Studies and int. comparison												
	**Localized cancers -** Node-negative cancers / screen-detected cancers (5)		Prevalent: >70% node negative Incident: <75%		Node negative cancers								% early stage	
	**Ductal carcinoma in situ (DCIS)** - % DCIS / all cancers (4)	Expert opinion	SL: 10%, DL 10-20%			Measured as inverse						In situ cancer detection rate		
	**Programme specificity** - % women without cancer with normal mammography (3)				False pos. rate									
	**Stage ≥2 cancers** – stage II+ cancers / screen-detected cancers (2)													
	**False negative rate** – false negatives / 1000 screens (2)													
	**Early recall** - % re-called at interval		AL: <1%, DL: 0%											

	shorter than normal (2)													
	**3rd reading** - % exams needing 3rd reading / all screens (1)													
	**Consistency in diagnosis**- diagnosis consistency (1)		Cancer kappa: AL 0.8, DL 0.9<br>DCIS kappa: AL 0.7, DL: 0.8											
Safety (4)	**Benign open biopsy or surgery rate** – benign biopsies or surgeries / 1000 screens (5)		Prevalent: <3.6 benign open biopsies / 1,000<br>Incident: <2.0 / 1,000			Ratio of benign: malignant surgery <1:4						Benign open biopsy and benign core biopsy		
	**Radiation dose** – measured once a week for standard mammogram (2)		Standard dose < 2.0 mGy											
	**False positive recall rate** – recalls not followed by cancer / 1000 screened (1)													
	**Mastectomy rate** – women with mastectomy / 1000 screened (1)													

Supplementary Table 2: Cervical cancer

Eight quality indicators identified from five organizations for cervical cancer screening with cervical smears, HPV testing or colposcopy. Definitions regroup common points from the listed articles, with important divergences noted. Grey-filled squares indicates that the quality indicator is present in that article with a similar definition to that listed. Targets are provided when available.

Category (number per category)	**Indicator** – definition (number of organizations using this indicator)	Level of evidence (Reported for 0/8 indicators)	Target (Reported for 3/8 indicators)	Organization (n=5), Year of publication				
				Europe Against Cancer Programme, 2010(14)	Czech screening program, 2017(15)	United Kingdom, National Health Service, 2021(16)	Canadian partnership against cancer, 2016(17)	United States, National Cancer Institute and others, 2015(18)
Adequacy (2)	**Specimen inadequacy** - % of cytology specimens deemed inadequate or unsatisfactory (3)		0.5 to 2% of tests inadequate, or 5th to 95th percentile					
	**Individual specimen adequacy (1 organization)** – specimens should have ≥5,000 squamous cells, with ≤75% of squamous cells obscured to be considered adequate, provided no abnormal cells are identified (1)							
Accuracy (6)	**Positive predictive value of abnormal results** - % of abnormal results found to have significant pathology on histology (3)		>90% for all abnormalities and >95% for high-grade abnormalities	% with CIN+ of those referred for colposcopy	For CIN2-positive results			
	**Pre-cancer detection rate** - # of precancerous lesions / 1000 women screened (3)				Rate of Abnormal smears and ASCUS			
	**Interval cancers** – % cancer diagnoses between 0.5 and 3 years after previous Pap test (2)							
	**Cytology – histology agreement** – proportion of high-grade pap tests with an abnormal histological outcome (1)		>65%					
	**Early-stage cancers** – % cancers detected at Stage I (1)							
	**Test specificity** - % women not referred to colposcopy / women without CIN+ (1)							

Supplementary Table 3: Colorectal cancer

Eighteen quality indicators from 14 organizations identified for colorectal cancer screening with colonoscopy or fecal occult blood tests (all types). Definitions regroup common points from the listed articles, with important divergences noted. Each ‘X’ indicates that the quality indicator is present in that article with a similar definition to that listed. Targets are provided when available.

Category	**Indicator** – definition (number of organizations using this indicator)	Level of evidence (Reported for 6/18 indicators)	Target (Reported for 14/18 indicators)	Organization (n=14), Year of publication													
				International Colorectal Cancer Screening Network, 2012(19)	European guidelines for quality assurance in CRC screening and	England, Bowel cancer screening programme, 2019(23)	Italy, Observatory for screening, 2010(24)	Netherlands Colonoscopy quality national program, 2019(25)	Spain, Cataluñacancer institute, 2013(26) and 2016(27)	Spain, Spanish Society of Gastroenterologists and Endoscopists, 2012(28)	UK key performance indicators for colonoscopy, 2021(29)	Asia-Pacific Consensus Recommendations, 2014(30)	Korea National Cancer Screening Program, 2018(31)	Canadian Partnership Against Cancer, 2017(32)	USA, AGA quality indicators for colonoscopy, 2015(33)	USA, ASGE/ACG, Quality indicators for colonoscopy and	USA, CDC demonstration project, 2013(36)
Adequacy (2)	**Adequate bowel preparation -** % adequate / colonoscopies (6)	Observ. Studies, clear benefit	≥85% or >90% of exams														
	**FOBT inadequacy rate** - % inadequate FOBT / tested population (6)		AL <3 %, DL < 1%											Target <5%			Adequate rate
Completeness (3)	**Cecal intubation rate** - % colonoscopies with photo taken of cecum (11)	Observ. Studies, clear benefit	AL >90%, DL ≥95%														
	**Scope withdrawal time –** average time (mins) to withdraw colonoscope when outcome normal (7)	Observ. Studies, unclear benefit	≥6 minutes														
	**Polyp removal/retrieval**		>90% polyps						As well as								

	**-** % polyps sent for pathology / all polyps (3)								>95% of polyps >10 mm								
Accuracy (8)	**Adenoma detection rate** – number with adenomas / endoscopies (10)	Observ. Studies, clear benefit	≥25% in mixed population			Screening vs diagnostic population				As well as adenomas / procedure				After FIT vs after gFOBT			
	**Cancer detection rate** – positive predictive value for cancer of positive tests (4)		≥2/1,000 people screened														
	**FOBT positivity rate** - % positive FOBT / tested population (4)		AL <6%, DL <5% first round														
	**Polyp detection rate** - number with polyps / endoscopies (2)		>65%														
	**CRC stage distribution**- % CRC in each stage		AL <30%, DL <20%				% stage III+		% stage I or II								
	**FOBT Positive predictive value** - % FIT with AA or CRC (1)																
	**High-grade neoplasia reported** - % of biopsies (1)		AL <5% screening colos, <10% diagnostic colos														
	**Post-colonoscopy CRC rate** - % CRCs after a negative colo (1)		<5% at 3 yrs														
Safety (5)	**Incidence of perforation -** % screening exams with perforation (4)	Observ. Studies, clear benefit	<1 / 1000														

	**Post-polypectomy bleeding** - % exams with polypectomy with bleeding (4)	Observ. Studies, clear benefit	<1%														
	**Adverse or unplanned events after colo** - % colos with adverse event w/in 30 days (4)								Compli-cation rate								
	**Mortality –** 30-day all-cause and colonoscopy-related mortality / tested population (1)																
	**Comfort score** - % colos with moderate-severe patient discomfort (1)																

Supplementary Table 4: Lung cancer

Seven quality indicators from three organizations identified for lung cancer screening with low-dose computed tomography (LDCT). Definitions regroup common points from the listed articles, with important divergences noted. Each 'X' indicates that the quality indicator is present in that article with a similar definition to that listed.

	**Indicator** – definition (number of organizations using this indicator)	Level of evidence (Reported for 0/7 indicators)	Target (Reported for 1/7 indicators)	Organization (n=3), Year		
				European Society of Radiology and European Respiratory Society, 2015(37)	England, National Health Service, 2020(38)	Canadian Partnership Against Cancer, 2018(39)
Accuracy (3)	**Early reassessment index** - % screens requiring any additional test or referral prior to the next routinely scheduled screen (1)					
	**Positive predictive value** – % diagnosed with lung cancer among those requiring early reassessment (1)					
	**Cancer detection rate –** of overall programme or among those biopsied / resected (1)					
Safety (4)	**Radiation dose** – Effective dose (3)		1-3 milli-sieverts			
	**Invasive procedure rate** - % participants who undergo an invasive procedure (1)					
	**Non-malignant surgical biopsy or resection rate** - # of surgical lung biopsies or resections with a non-malignant result / 1,000 screens (1)					
	**30-day mortality rate after surgical procedure** - % who died within 30 days of among participants who underwent a surgical procedure (1)					

References

1. Muratov S, Canelo-Aybar C, Tarride JE, Alonso-Coello P, Dimitrova N, Borisch B, et al. Monitoring and evaluation of breast cancer screening programmes: selecting candidate performance indicators. BMC Cancer. 2020;20(1):795.
2. Goossens M, De Brabander I, De Greve J, Van Ongeval C, Martens P, Van Limbergen E, et al. Flemish breast cancer screening programme: 15 years of key performance indicators (2002-2016). BMC Cancer. 2019;19(1):1012.
3. Langagergaard V, Garne JP, Vejborg I, Schwartz W, Bak M, Lernevall A, et al. Existing data sources for clinical epidemiology: the Danish Quality Database of Mammography Screening. Clin Epidemiol. 2013;5:81-8.
4. Cohen SL, Blanks RG, Jenkins J, Kearins O**.** Role of performance metrics in breast screening imaging - where are we and where should we be? Clin Radiol. 2018;73(4):381-8.
5. Public Health England 2021;Pages. Accessed at National Health Service at https://www.gov.uk/government/publications/breast-screening-consolidated-programme-standards/nhs-breast-screening-programme-screening-standards-valid-for-data-collected-from-1-april-2021 on Dec 21 2021.
6. Fitzpatrick PE, Greehy G, Mooney MT, Flanagan F, Larke A, Connors A, et al. Evolution of the National Breast Screening Programme in Ireland: Two-year interval analysis (2004-2013) of BreastCheck. J Med Screen. 2018;25(4):191-6.
7. The National Breast Screening Programme**.** Guidelines for Quality Assurance in Mammography Screening. Dublin: National Screening Service; 2015.
8. Giordano L, Castagno R, Giorgi D, Piccinelli C, Ventura L, Segnan N, et al. Breast cancer screening in Italy: evaluating key performance indicators for time trends and activity volumes. Epidemiol Prev. 2015;39(3 Suppl 1):30-9.
9. Loy EY, Molinar D, Chow KY, Fock C**.** National Breast Cancer Screening Programme, Singapore: evaluation of participation and performance indicators. J Med Screen. 2015;22(4):194-200.
10. Pan; H-B, Hsu; G-C, Liang; H-L, Chou; C-P, Wang; Y-C, Lee; S-K, et al. Assessing an Emerging Nationwide Population-based Mammography Screening Program in Taiwan. Journal of Radiological Science. 2011;36(1):3-9.
11. The Canadian Partnership Against Cancer**.** Guidelines for Monitoring Breast Cancer Screening Program Performance (3rd edition). Toronto: Canadian Partnership Against Cancer; 2013.
12. Rauscher GH, Murphy AM, Orsi JM, Dupuy DM, Grabler PM, Weldon CB**.** Beyond the mammography quality standards act: measuring the quality of breast cancer screening programs. AJR Am J Roentgenol. 2014;202(1):145-51.
13. Sprague BL, Arao RF, Miglioretti DL, Henderson LM, Buist DS, Onega T, et al. National Performance Benchmarks for Modern Diagnostic Digital Mammography: Update from the Breast Cancer Surveillance Consortium. Radiology. 2017;283(1):59-69.
14. Arbyn M, Anttila A, Jordan J, Ronco G, Schenck U, Segnan N, et al. European Guidelines for Quality Assurance in Cervical Cancer Screening. Second Edition—Summary Document. Annals of Oncology. 2010;21(3):448-58.
15. Majek O, Duskova J, Dvorak V, Bekova A, Klimes D, Blaha M, et al. Performance indicators in a newly established organized cervical screening programme: registry-based analysis in the Czech Republic. Eur J Cancer Prev. 2017;26(3):232-9.
16. Public Health England 2021;Pageshttps://www.gov.uk/government/publications/cervical-screening-programme-standards/cervical-screening-programme-standards-valid-for-data-collected-from-1-april-2018 on Dec 22 2021.

17. The Canadian Partnership Against Cancer**.** Cervical cancer screening in canada: Monitoring & evaluation of quality indicators. Toronto: Canadian Partnership Against Cancer; 2016.
18. The Bethesda System for Reporting Cervical Cytology. Cham: Springer International Publishing AG; 2015.
19. Benson VS, Atkin WS, Green J, Nadel MR, Patnick J, Smith RA, et al. Toward standardizing and reporting colorectal cancer screening indicators on an international level: The International Colorectal Cancer Screening Network. Int J Cancer. 2012;130(12):2961-73.
20. von Karsa L, Patnick J, Segnan N**.** European guidelines for quality assurance in colorectal cancer screening and diagnosis. First Edition--Executive summary. Endoscopy. 2012;44 Suppl 3:SE1-8.
21. Valori R, Rey JF, Atkin WS, Bretthauer M, Senore C, Hoff G, et al. European guidelines for quality assurance in colorectal cancer screening and diagnosis. First Edition--Quality assurance in endoscopy in colorectal cancer screening and diagnosis. Endoscopy. 2012;44 Suppl 3:SE88-105.
22. Moss S, Ancelle-Park R, Brenner H, International Agency for Research on C**.** European guidelines for quality assurance in colorectal cancer screening and diagnosis. First Edition--Evaluation and interpretation of screening outcomes. Endoscopy. 2012;44 Suppl 3:SE49-64.
23. Public Health England 2021;Pageshttps://www.gov.uk/government/publications/bowel-cancer-screening-programme-standards/bowel-cancer-screening-programme-standards-valid-for-data-collected-from-1-april-2018 on Dec 22 2021.
24. Zorzi M, Baracco S, Fedato C, Grazzini G, Naldoni C, Sassoli de Bianchi P, et al. Screening for colorectal cancer in Italy: 2008 survey. Epidemiol Prev. 2010;34(5-6 Suppl 4):53-72.
25. Bronzwaer MES, Depla A, van Lelyveld N, Spanier BWM, Oosterhout YH, van Leerdam ME, et al. Quality assurance of colonoscopy within the Dutch national colorectal cancer screening program. Gastrointest Endosc. 2019;89(1):1-13.
26. Binefa G, Garcia M, Mila N, Rodriguez L, Rodriguez-Moranta F, Guardiola J, et al. Colonoscopy quality assessment in a mass population screening programme based on faecal occult blood test. Rev Esp Enferm Dig. 2013;105(7):400-8.
27. Binefa G, Garcia M, Mila N, Fernandez E, Rodriguez-Moranta F, Gonzalo N, et al. Colorectal Cancer Screening Programme in Spain: Results of Key Performance Indicators After Five Rounds (2000-2012). Sci Rep. 2016;6:19532.
28. Jover R, Herraiz M, Alarcon O, Brullet E, Bujanda L, Bustamante M, et al. Clinical practice guidelines: quality of colonoscopy in colorectal cancer screening. Endoscopy. 2012;44(4):444-51.
29. Rees CJ, Thomas Gibson S, Rutter MD, Baragwanath P, Pullan R, Feeney M, et al. UK key performance indicators and quality assurance standards for colonoscopy. Gut. 2016;65(12):1923-9.
30. Sung JJ, Ng SC, Chan FK, Chiu HM, Kim HS, Matsuda T, et al. An updated Asia Pacific Consensus Recommendations on colorectal cancer screening. Gut. 2015;64(1):121-32.
31. Min JK, Cha JM, Cho YK, Kim JH, Yoon SM, Im JP, et al. Revision of Quality Indicators for the Endoscopy Quality Improvement Program of the National Cancer Screening Program in Korea. Clin Endosc. 2018;51(3):239-52.
32. The Canadian Partnership Against Cancer, 2017;Pageshttps://s22457.pcdn.co/wp-content/uploads/2019/01/Colorectal-Screening-Monitoring-Report-2014-EN.pdf on Dec 22 2021.
33. Rex DK, Schoenfeld PS, Cohen J, Pike IM, Adler DG, Fennerty MB, et al. Quality indicators for colonoscopy. Gastrointestinal Endoscopy. 2015;81(1):31-53.
34. Rizk MK, Sawhney MS, Cohen J, Pike IM, Adler DG, Dominitz JA, et al. Quality indicators common to all GI endoscopic procedures. Gastrointest Endosc. 2015;81(1):3-16.

35. Robertson DJ, Lee JK, Boland CR, Dominitz JA, Giardiello FM, Johnson DA, et al. Recommendations on fecal immunochemical testing to screen for colorectal neoplasia: a consensus statement by the US Multi-Society Task Force on colorectal cancer. Gastrointest Endosc. 2017;85(1):2-21 e3.
36. Nadel MR, Royalty J, Shapiro JA, Joseph D, Seeff LC, Lane DS, et al. Assessing screening quality in the CDC's Colorectal Cancer Screening Demonstration Program. Cancer. 2013;119 Suppl 15:2834-41.
37. Kauczor HU, Bonomo L, Gaga M, Nackaerts K, Peled N, Prokop M, et al. ESR/ERS white paper on lung cancer screening. Eur Radiol. 2015;25(9):2519-31.
38. NHS England 2020;Pageshttps://www.england.nhs.uk/wp-content/uploads/2019/02/targeted-screening-for-lung-cancer-quality-assurance-standard.pdf on Dec 22 2021.
39. The Canadian Partnership Against Cancer 2018;Pageshttps://s22457.pcdn.co/wp-content/uploads/2019/01/Lung-Companion-Implementation-Resource-2018-EN.pdf on Dec 22 2021.
